# Integrated genomic analysis of mitochondrial RNA processing in human cancers

**DOI:** 10.1186/s13073-017-0426-0

**Published:** 2017-04-18

**Authors:** Youssef Idaghdour, Alan Hodgkinson

**Affiliations:** 1 Biology Program, Division of Science and Mathematics, New York University Abu Dhabi, PO Box 129188, Abu Dhabi, United Arab Emirates; 2 Department of Medical and Molecular Genetics, Guy’s Hospital, King’s College London, London, SE1 9RT UK

**Keywords:** Transcriptomics, Mitochondria, RNA processing, Mitochondrial tRNA, Cancer

## Abstract

**Background:**

The mitochondrial genome is transcribed as continuous polycistrons of RNA containing multiple genes. As a consequence, post-transcriptional events are critical for the regulation of gene expression and therefore all aspects of mitochondrial function. One particularly important process is the m^1^A/m^1^G RNA methylation of the ninth position of different mitochondrial tRNAs, which allows efficient processing of mitochondrial mRNAs and protein translation, and de-regulation of genes involved in these processes has been associated with altered mitochondrial function. Although mitochondria play a key role in cancer, the status of mitochondrial RNA processing in tumorigenesis is unknown.

**Methods:**

We measure and assess mitochondrial RNA processing using integrated genomic analysis of RNA sequencing and genotyping data from 1226 samples across 12 different cancer types. We focus on the levels of m^1^A and m^1^G RNA methylation in mitochondrial tRNAs in normal and tumor samples and use supervised and unsupervised statistical analysis to compare the levels of these modifications to patient whole genome genotypes, nuclear gene expression, and survival outcomes.

**Results:**

We find significant changes to m^1^A and m^1^G RNA methylation levels in mitochondrial tRNAs in tumor tissues across all cancers. Pathways of RNA processing are strongly associated with methylation levels in normal tissues (*P* = 3.27 × 10^−31^), yet these associations are lost in tumors. Furthermore, we report 18 gene-by-disease-state interactions where altered RNA methylation levels occur under cancer status conditional on genotype, implicating genes associated with mitochondrial function or cancer (e.g., *CACNA2D2*, *LMO2*, and *FLT3*) and suggesting that nuclear genetic variation can potentially modulate an individual’s ability to maintain unaltered rates of mitochondrial RNA processing under cancer status. Finally, we report a significant association between the magnitude of methylation level changes in tumors and patient survival outcomes.

**Conclusions:**

We report widespread variation of mitochondrial RNA processing between normal and tumor tissues across all cancer types investigated and show that these alterations are likely modulated by patient genotype and may impact patient survival outcomes. These results highlight the potential clinical relevance of altered mitochondrial RNA processing and provide broad new insights into the importance and complexity of these events in cancer.

**Electronic supplementary material:**

The online version of this article (doi:10.1186/s13073-017-0426-0) contains supplementary material, which is available to authorized users.

## Background

The role of mitochondria in cancer has long been controversial. Although mitochondria are essential for tumor cell growth [1–3], many lines of evidence indicate that altered mitochondrial bioenergetics are required for tumor initiation and persistence. First, the up-regulation of anaerobic energy production via glycolysis, the so-called Warburg effect, is well documented and recognized as a hallmark of cancer [4, 5]. Second, mutations in nuclear-encoded mitochondrial genes have been identified in patients with cancer, with links to the disease well established in some cases [6]. Third, increased numbers of mutations are consistently found in the mitochondrial genomes of tumor cells compared to normal samples [7–9]. These mutations may merely tag carcinogenesis, but whether other genetic properties of mitochondrial genomes are important in tumorigenesis remains one of the important unanswered questions in cancer biology.

In line with this, recent studies have looked beyond mitochondrial DNA mutations to consider other important genetic processes. Mitochondrial copy number has been found to vary between paired normal and tumor samples [10, 11], mitochondrial biogenesis is often altered in cancer cells [6, 10], and recent work has suggested that mitochondrial RNA transcripts may accumulate differently in cancer tissues [12]. The idea that post-transcriptional processing of the mitochondrial transcriptome may be altered in cancer is intriguing. Mitochondrial RNA is transcribed as continuous polycistrons, which are then processed under the “punctuation model”, whereby tRNAs that intersperse mRNAs are targeted for modification and cleavage by nuclear-encoded proteins [13–16]. The polycistronic nature of mitochondrial transcription means that post-transcriptional events are particularly important: knockdown of RNA processing enzymes influences mitochondrial mRNA and protein levels, and mitochondrial function [17] and the level of m^1^A and m^1^G post-transcriptional methylation at the ninth position of mitochondrial tRNAs (p9 sites) can potentially influence downstream metabolic phenotypes [18]. Indeed, p9 site methylation is thought to influence the correct folding of mitochondrial tRNAs, thus affecting the rate of cleavage within the polycistronic transcript and potentially impacting upon their downstream roles in protein translation [19–21].

There are several reasons to believe that altered processing of mitochondrial RNA (which we now use to denote all processing events that occur after transcription of the mitochondrial polycistronic strand, including nucleotide modifications, base additions, and strand cleavage events) may be involved in cancer. For example, mutations within the mitochondrial processing enzyme RNase Z were found to be segregating with prostate cancer incidence in human pedigrees [22], and mutations within mitochondrial tRNAs, which are heavily post-transcriptionally modified, have been previously linked with cancer [23]. Here, we assess whether mitochondrial RNA processing is altered in cancer by analyzing RNA sequencing data from 1226 paired normal and tumor samples across 12 cancer types from The Cancer Genome Atlas (TCGA). We find significant and consistent signatures of increased mitochondrial tRNA p9 site methylation in tumor tissues compared to paired adjacent normal samples that appears to be coupled with major deregulation of nuclear RNA processing genes. Furthermore, we find evidence of context-specific SNPs that are associated with methylation levels in tumor but not normal samples (genotype-by-disease-state interactions), and we observe a significant relationship between the magnitude of change in mitochondrial tRNA p9 site methylation in tumor tissues and the survival outcome of patients with kidney renal clear cell carcinoma, thus highlighting the potential clinical relevance of these events in tumorigenesis.

## Methods

### RNA sequencing data

Raw sequencing files (fastq format) were obtained from TCGA through the CGHub repository via dbGaP accession number phs000178.v9.p8 [24, 25] for 12 cancer types where at least 25 paired tumor and adjacent normal samples were available (we do not use matched blood normal samples). These included breast invasive carcinoma (BRCA), colon adenocarcinoma (COAD), head and neck squamous cell carcinoma (HNSC), kidney chromophobe (KICH), kidney renal clear cell carcinoma (KIRC), kidney renal papillary cell carcinoma (KIRP), liver hepatocellular carcinoma (LIHC), lung adenocarcinoma (LUAD), lung squamous cell carcinoma (LUSC), prostate adenocarcinoma (PRAD), stomach adenocarcinoma (STAD), and thyroid carcinoma (THCA). In total, we obtained 1226 RNA sequencing datasets for analysis.

Sequencing reads were trimmed for adaptor sequences, terminal bases with quality lower than 20, and poly(A) tails of five nucleotides or greater before being aligned to a reference genome (1000G GRCh37 reference, which contains the mitochondrial rCRS NC_012920.1) with STAR 2.51a [26], using default parameters, two-pass mapping, and version 19 of the Gencode gene annotation. Careful attention was paid to minimize the likelihood of incorrectly placed reads, particularly those associated with NUMT sequences. To achieve this, a stringent filtering pipeline was applied, as we previously demonstrated [18], focusing only on properly paired and uniquely mapped reads.

### Gene expression levels

To calculate transcript abundances, we used HTseq [27] with default parameters, the “intersection-nonempty” model, and Gencode gene annotation file v19. Raw counts were then converted to transcripts per million (TPM). Within the TPM calculation, for mitochondrial genes the total number of fragments mapping to the mitochondrial transcriptome was used to normalize for library size. Since the total amount of mitochondrial RNA in each sample is influenced by both mitochondrial copy number and polycistronic transcription rate, normalizing the data in this way controls for these two factors and allows us to focus on variation in gene expression driven by processing of the polycistronic strand. For nuclear genes, the total library size was used. TPM scores were then log_10_transformed and median normalized. Principal component analysis and distribution analysis were used to identify outlier samples. Samples greater than three standard deviations from the mean in any of the first three principal components were deemed outliers. All samples paired with these outliers were also removed from subsequent analysis, resulting in a set of 1196 samples across all cancers. Distribution analysis shows that samples within each cancer type had similar distributions, suggesting that variations in the data due to technical reasons are minimal.

### Methylation levels at tRNA p9 sites

Previous studies have highlighted that sequencing mismatches observed in RNA sequencing data at particular positions in the mitochondrial genome represent post-transcriptional modification events [17, 18, 28]. The assumption behind this approach is that chemical modifications of RNA either act as a road-block to the reverse transcription enzyme during library preparation or cause the enzyme to mis-incorporate nucleotides, resulting in sequencing errors [29]. Recent work by Mercer et al. [28], Sanchez et al. [17], and ourselves [18] has shown that sequence mismatches occur at a high rate at the ninth position of different mitochondrial tRNAs, which are positions that are known to be post-transcriptionally methylated, and subsequent experiments by ourselves [18] have shown that the proportion of mismatches at these sites is systematic and repeatable across replication experiments. Recently, new sequencing methods have confirmed the presence of post-transcriptional methylation events at 19/22 p9 sites by comparing RNA sequencing data from samples that have been treated with demethylation enzymes against matched untreated samples [30]. Within this work, the general quantitative nature of using mismatch and strand termination events as a proxy for post-transcriptional modification was also shown, and although the ratio of these events does not perfectly match post-transcriptional modification levels (since the reference allele is sometimes incorporated), the two levels are highly similar. Under this model, we inferred the level of p9 site methylation as the proportion of non-reference alleles for the 11 positions identified as undergoing post-transcriptional methylation in our previous study [18] (positions 585, 1610, 4271, 5520, 7526, 8303, 9999, 10413, 12174, 12246, and 14734 in the mitochondrial genome) using samtools v1.2 *mpileup* [31] with default parameters to generate allele count files. It is important to note that these p9 positions in the mitochondrial genome have previously been shown not to overlap with known variants in NUMT sequences in the human reference using a careful and stringent mapping and filtering strategy [18].

Within each cancer type (12 in total), we compare methylation levels between normal and tumor samples for each of the 11 p9 sites separately using Wilcoxon signed rank tests, using only those sites where both tumor and paired normal samples had at least 20× coverage. Across all samples, the average coverage at p9 sites is ~600×. As an example, inferred methylation levels for the 11 p9 sites for KIRC are shown in Additional file 1: Figure S1. In order to control for any biases in coverage, we repeated the analysis after resampling sequencing reads within each individual and at each site to the lowest coverage found at that site in either the normal or tumor sample. To directly compare the levels of p9 methylation across cancers, we standardized rates at each p9 site within each cancer by dividing the proportion of mismatches by the maximum value observed across normal and tumor samples at each site. This normalization ensures that methylation levels are on the same scale across sites and cancers, yet variation is maintained across samples. To compare the levels of p9 methylation with cleavage rates at the 5′ end of mitochondrial tRNAs, we calculated the proportion of reads that started or ended either side of the position 9 bp upstream of the p9 site compared to all reads covering that position. We considered only sites with at least 20 individuals with 20× coverage at both cleavage and p9 positions and used Spearman rank correlation tests. For comparisons to mitochondrial gene expression, we performed Spearman rank correlation tests for each p9 site and within each cancer for normal and tumor samples separately.

To ensure that confounding factors are not influencing our results, we tested whether a number of features were associated with p9 site methylation levels. In general, since paired normal and tumor samples originate from the same individual, many phenotypes will be identical between sample pairs. However, to ensure that age and sex are not affecting the results, we tested whether these factors are correlated with methylation levels within either normal or tumor samples for individuals that we had genotyping data for; in all cases we find no significant relationships (*P* > 0.05 after Bonferroni correction). To test the impact of varying tumor purity on our results we obtained measures of tumor purity and histological estimates of stromal cells and infiltrating lymphocytes from Yoshihara et al. [32] and compared these measures to changes in p9 site methylation levels between matched normal and tumor samples within each cancer type using Spearman rank correlations. In total, only one comparison out of 132 (11 p9 sites in 12 different cancers) was significant after Bonferroni correction (site 7526, stromal infiltrate in individuals with PRAD, *P* = 0.00013), suggesting that tumor purity is not driving the observed changes in tumor samples.

### Differential expression and cross-correlations with nuclear gene expression

We evaluated the magnitude and significance of differential expression of transcripts of nuclear-encoded mitochondrial RNA-binding proteins [33] using analysis of variance and Bonferroni thresholds to infer statistical significance. In total, 99 transcripts out of 107 listed in Wolf and Mootha [33] were deemed expressed in BRCA and KIRC datasets (100 transcripts in THCA). Two-way clustering of gene expression data of the full set of genes encoding mitochondrial RNA-binding proteins was generated using Ward’s method in JMP Genomics 8.0 (SAS Institute). To investigate the relationships between nuclear gene expression traits and p9 site methylation level, we performed an unbiased genome-wide association between methylation level at 11 p9 sites and 16,736 expression traits in the BRCA dataset. We calculated Spearman correlation across all individuals in each sample type. The significance of correlations was assessed by correcting for multiple testing, resulting in a Bonferroni threshold of 3 × 10^−6^. In the main text we present results for BRCA; however, we see the same general trends for THCA and KIRC as significant associations in normal tissue are altered in tumor samples (Additional file 1: Figure S2). Gene set enrichment analysis was performed using the Core Analysis Workflow implemented in the Ingenuity Pathway Analysis package to measure the likelihood that the association between nuclear genes whose expression was significantly associated with p9 site methylation level and a given process or pathway is due to random chance. The *P* value is calculated using the right-tailed Fisher exact test that takes into consideration the number of focus genes that participate in the process in question and the total number of genes that are known to be associated with that process in the human reference set. The same analysis parameters were used for BRCA, KIRC, and THCA (Additional file 1: Table S1).

### Statistical interaction effects

Where available, we downloaded birdseed genotype files generated from Affymetrix Genome-wide Human SNP arrays (6.0) for all individuals for which we had RNA sequencing data. Samples that did not pass TCGA quality control were not used and, in total, data were available for 569 individuals. For each cancer type, we filtered out genotypes with birdseed quality scores below 0.1 and kept SNPs in Hardy–Weinberg equilibrium (*P* > 0.001). Since sample sizes within cancers were generally small, we converted minor homozygote alleles to heterozygotes (dominant model). SNPs with minor allele frequency (MAF) <5% were removed and we then ran a quantitative trait model (GxE) in plink 1.07 [34], using the levels of p9 methylation at sites where paired normal and tumor samples had at least 20× coverage. Within these tests, the levels of p9 site methylation were used as the quantitative trait and sample type (normal or tumor) as the environment, and regression coefficients were compared between normal and tumor association tests to generate a *P* value for the interaction term. In order to ensure robust findings, we considered only sites that had data for at least 40 individuals. QQ plots for p9 sites and cancers showing significant interaction effects are shown in Additional file 1: Figure S3. After identifying SNPs that passed genome-wide significance, we visually inspected data plots and made sure that the uncovered associations are not driven by outliers. SNP annotations were taken from the Affymetrix annotation file associated with the array.

### Survival analysis

We obtained patient survival data from TCGA and performed survival analysis in R using the package “Survival”. We limited analysis to cancers for which we had RNA sequencing data for at least 50 individuals, with a death rate of 25% (KIRC and LUAD). Censoring was limited to 60 months, since most events happen during this time. Cox proportional hazards tests were used to model survival as a function of changes in p9 site methylation levels in tumor versus normal samples. For significant associations, we tested Schoenfeld residuals to ensure that the proportional hazards assumption was being met (*P* > 0.05 in all cases). To calculate meaningful hazard ratios, the tests were repeated after binning changes in the levels of p9 site methylation into two equal sized groups.

## Results

### Post-transcriptional changes in tumor cells

**Fig. 1:**
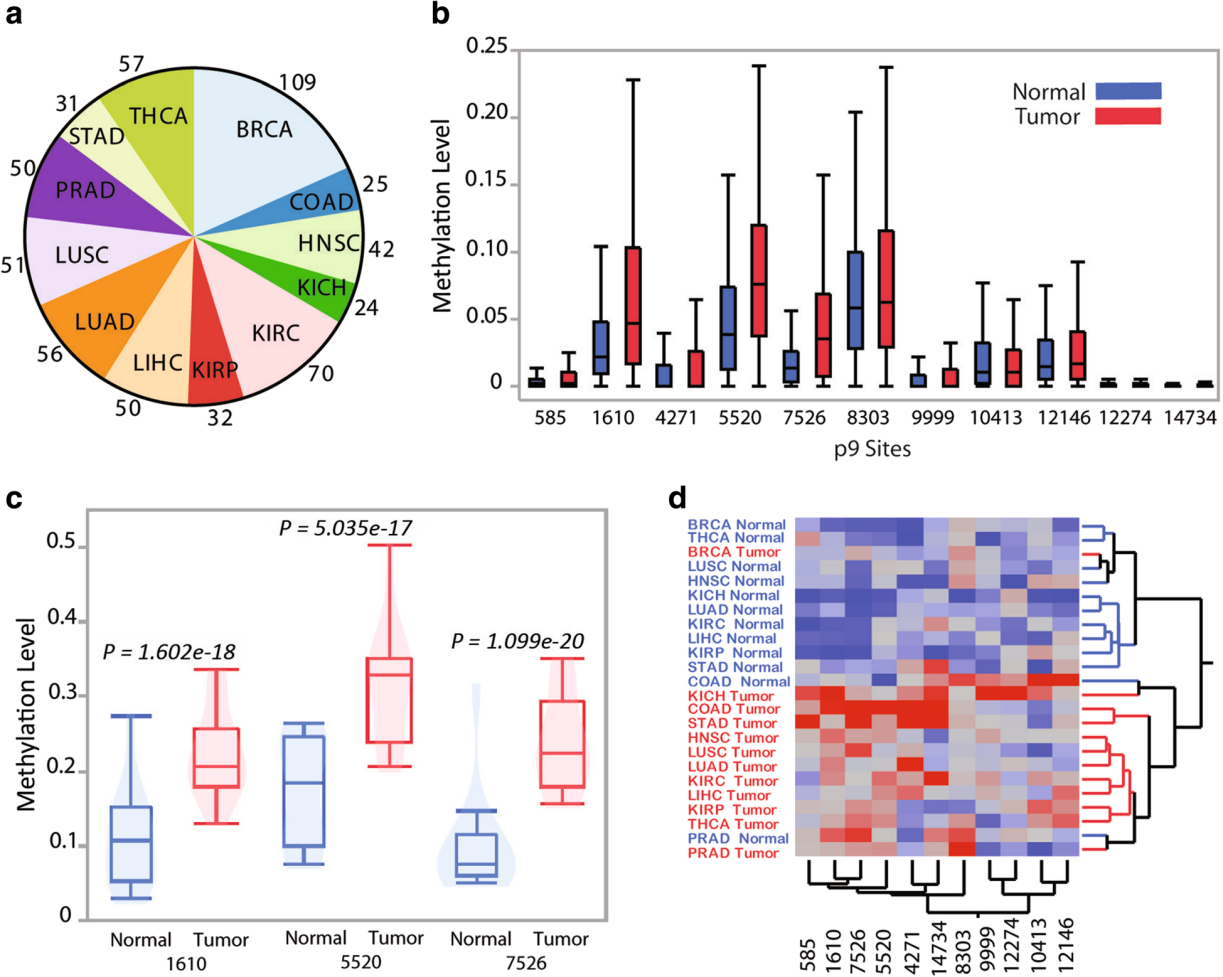
Methylation differences between paired tumor and normal samples at tRNA p9 sites within mitochondrial tRNAs. **a** Number of normal–tumor pairs for each cancer type, including breast invasive carcinoma (*BRCA*), colon adenocarcinoma (*COAD*), head and neck squamous cell carcinoma (*HNSC*), kidney chromophobe (*KICH*), kidney renal clear cell carcinoma (*KIRC*), kidney renal papillary cell carcinoma (*KIRP*), liver hepatocellular carcinoma (*LIHC*), lung adenocarcinoma (*LUAD*), lung squamous cell carcinoma (*LUSC*), prostate adenocarcinoma (*PRAD*), stomach adenocarcinoma (*STAD*) and thyroid carcinoma (*THCA*). **b** Observed methylation levels for all 11 p9 sites within KIRC, split into normal and tumor. A similar plot showing all data points is shown in Additional file 1: Figure S1. **c** Standardized methylation levels split into normal and tumor for all cancers combined for p9 sites 1610, 5520, and 7526. **d** Two-way hierarchical clustering of mean standardized methylation levels of all 11 p9 sites for both normal and tumor samples across all cancer types

To study the patterns of mitochondrial RNA processing in human cancers we mapped and filtered raw RNA sequencing data from 1226 samples from matched tumor–normal pairs across 12 cancer types from TCGA (Fig. 1a). Using these data we inferred the level of m^1^A and m^1^G post-transcriptional methylation occurring at 11 functionally important positions within mitochondrial tRNAs (the ninth position of 11 different tRNAs, as identified in [18], henceforth referred to as p9 sites) by using the proportion of mismatches observed at these positions (referred to as “p9 site methylation levels” throughout; see “Methods”), in line with approaches taken by previous studies [17, 18, 28]. For each of the 11 p9 sites and within each of the 12 cancers, we then compared the level of p9 site methylation observed between paired normal and tumor samples (11 × 12 = 132 comparisons in total).

In total, 50/132 comparisons show significant differences between normal and tumor tissues at a 5% significance level (Wilcoxon signed-rank tests) and 42 of these comparisons show increases in the observed levels of p9 site methylation in cancer tissue (Fig. 1b for examples showing all 11 p9 sites in KIRC; Additional file 1: Table S2). After applying Bonferroni correction (within each cancer type, *P* < 0.0045), 23 comparisons remain significant, 22 of which show increases of the levels of p9 site methylation in cancer tissues. Resampling sequencing reads to the same depth in paired normal and tumor samples (thus accounting for potential biases in sequencing coverage) gives very similar results (Additional file 1: Table S3; see “Methods”). These observations strongly suggest a widespread increase in the level of p9 site methylation of mitochondrial tRNAs in tumor tissue in multiple cancer types.

Next, we investigated whether the observed differences in the levels of p9 site methylation are a general trend in cancer. For each p9 site, we standardized the data within each cancer type (thus maintaining cancer-associated patterns in methylation levels) and tested differences between tumor and normal pairs across all cancer types combined. In total, five out of the 11 p9 sites show highly significant differences (*P* < 0.0045; Additional file 1: Table S4; Fig. 1c for p9 sites 1610, 5520, and 7526). Following this, we averaged the level of p9 site methylation by group and found that, strikingly, two-way hierarchical clustering using all p9 site data from 24 cancer sample-type sets revealed how groups cluster largely by sample type (normal or tumor) with the exception of BRCA tumor, COAD normal, and PRAD normal not clustering within their respective sample type (Fig. 1d). These results suggest that altered processing of mitochondrial RNA is a consistent trend across multiple cancer types.

To infer the impact of changes at sites and cancers where we observe significant differences (at *P* < 0.05), we compared the levels of p9 site methylation across both normal and tumor samples combined with the levels of cleavage occurring at the 5′ end of each respective tRNA, which we measured as the proportion of sequencing reads starting or ending either side of this position. As a control, we considered the level of cleavage at a further 9 bp upstream from each p9 site. Methylation levels significantly correlate with cleavage rates for 9/43 comparisons after Bonferroni correction (*P* < 0.001, versus one significant correlation for the control) and for 16/43 comparisons at a 5% significance level, with all significant correlations being in the positive direction (Additional file 1: Table S5). Next, for the same sites we tested whether p9 site methylation levels are associated with tRNA expression levels across normal and cancer samples combined. To calculate tRNA expression, we first normalized the coverage at each site by dividing by the total number of reads mapping to the mitochondria (following the approach of Stewart et al. [12]), and then averaged the rate across each respective tRNA. In total, at 24/50 p9 sites the level of methylation is correlated with tRNA expression levels (*P* < 0.05, seven positive and 17 negative; Additional file 1: Table S6). Finally, we considered the influence of changes in p9 site methylation in cancer on mitochondrial coding gene expression: considering normal samples separately, the levels of p9 methylation significantly correlate with mitochondrial gene expression in 25 comparisons across cancer types (Additional file 2: Table S7; Spearman rank *P* < 0.05 after Bonferroni correction within cancer type, mostly negatively correlated with *MTCO3*, *MTCO2*, and *MTCO1* abundance and positively correlated with *MTND2* abundance), yet in tumor samples these relationships break down and only two pairwise comparisons are significant. In all, these analyses point to a link between p9 site methylation levels and other RNA processing events taking place after transcription of the mitochondrial polycistronic transcript.

### Nuclear transcriptional signatures associated with changes in mitochondrial RNA processing

**Fig. 2:**
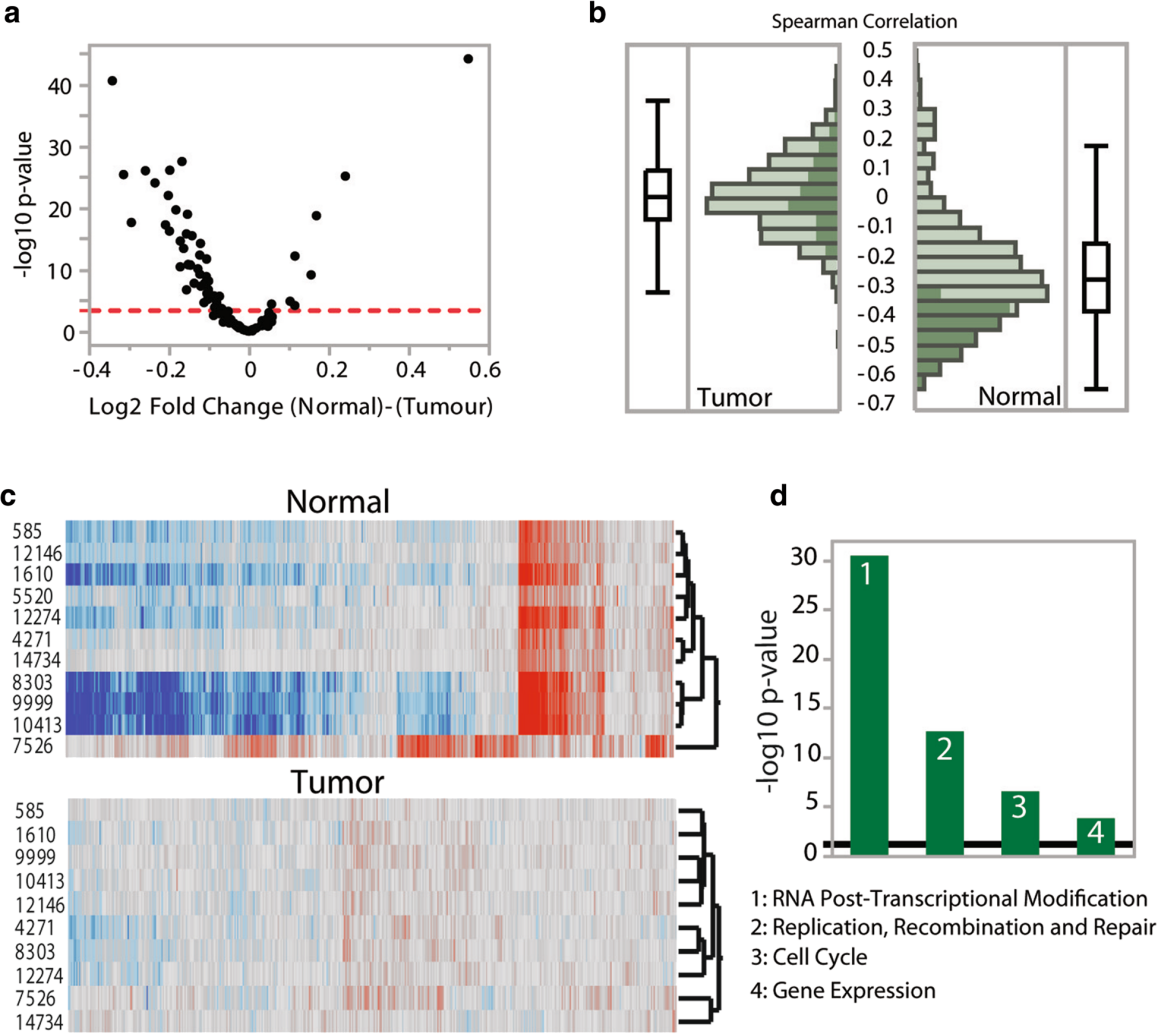
Differential expression and correlations between the levels of tRNA p9 methylation and nuclear gene expression in BRCA. **a** Volcano plot of statistical significance (shown as the negative logarithm of the *P* value on the *y-axis*) versus magnitude of differential gene expression (shown as the log base 2 of magnitude of mean expression difference on the *x-axis*) of 99 genes encoding mtRNA-binding proteins. The *dashed line* indicates Bonferroni statistical significance. **b** Distribution of Spearman correlations between gene expression levels of the 99 genes and the levels of methylation at p9 site 10413. Associations that are significant at Bonferroni threshold in normal samples are highlighted with the *dark green* color. **c** Two-way clustering of Spearman correlations of expression levels of 16,736 genes (*columns*) and methylation level at 11 p9 sites (*rows*) in the BRCA dataset. Correlation values are visualized using a *red-*to*-gray*-to-*blue* color theme (values range from 0.62 to −0.66). **d** Ingenuity pathway enrichment analysis of nuclear genes whose expression levels are associated with the levels of methylation at p9 site 10413 in the BRCA dataset. The top four categories are shown and the *P* value represents the most significant biological sub-function within each category

Mitochondrial RNA transcription and processing, like many other molecular processes taking place in mitochondria, is under strong nuclear control. As such, we tested the hypothesis that the expression of nuclear-encoded genes involved in mitochondrial RNA processing is altered in tumors by performing differential expression analysis. Raw RNA sequencing data were aligned, filtered, and normalized as detailed in the “Methods” and expression data for 99 mitochondrial RNA-binding proteins (as listed in [33]) were retrieved and compared between normal and tumor samples. In this section we report results for BRCA only, since this is the cancer type for which we have the most paired samples (>100) and thus the most power; however, we also see the same broad trends in other cancer types with the next largest sample sizes (see “Methods”). In total, we detected 55 genes differentially expressed at Bonferroni significance (Fig. 2a) and the heatmap of all 99 gene expression traits surveyed shows that samples cluster largely by sample type (normal or tumor; Additional file 1: Figure S4).

Following this, we tested for associations between the expression levels of the 99 nuclear-encoded factors and the levels of p9 site methylation. We performed cross-correlation analysis (Spearman rank) across all individuals for all possible 1089 p9 site methylation level–nuclear gene expression trait pairs in normal and tumor samples separately. The test revealed significant associations for eight of the 11 p9 sites in normal samples and the total cumulative number of Bonferroni-significant associations was 369 across all eight p9 sites (*P* < 0.0005; Fig. 2b for distributions of coefficients; variance explained ranges between −0.64 and 0.47). In sharp contrast, no significant associations were detected in tumor samples (Fig. 2b), indicating major deregulation of these processes in cancer. A two-way hierarchical clustering heatmap of the full correlation matrix (Additional file 1: Figure S5) shows the consistency of the associations across different p9 sites in normal samples, whereas these associations are highly perturbed under cancer status.

Next, we identified cell-wide processes associated with mitochondrial RNA processing by performing global cross-correlation analysis between the expression levels of all nuclear genes and the levels of methylation at each p9 position using the same strategy as outlined above and using a Bonferroni corrected *P* value threshold of 3 × 10^−6^ (0.05/16,736). In normal samples, the test revealed an average of 2311 significant associations for eight of the 11 p9 sites (Fig. 2c; Additional file 1: Table S8; three sites show no significant associations; variance explained ranges between −0.66 and 0.62). To investigate the functional characteristics of nuclear genes whose expression levels are associated with the levels of methylation at the p9 site showing the strongest signal in normal cells (p9 site 10413 and 6061 genes) we used Ingenuity Pathway Analysis. We find that “RNA post-transcriptional modification” is the top and most highly enriched molecular and cellular function category with five significantly enriched sub-functions (*P* value range 2.77 × 10^−6^ to 3.27 × 10^−31^; Fig. 2d; “Processing of RNA” was the most enriched sub-function), supporting the idea that many nuclear genes play a role in mitochondrial RNA processing. We also observe similar results for other cancer types and at other p9 sites (see “Methods”). However, we again find striking differences in tumor samples, where we detect only two significant associations across all p9 sites, pointing to major deregulation of nuclear-associated mitochondrial RNA processing in cancer (Additional file 1: Figure S6).

### Joint action of genotype and cancer state on post-transcriptional methylation

**Fig. 3:**
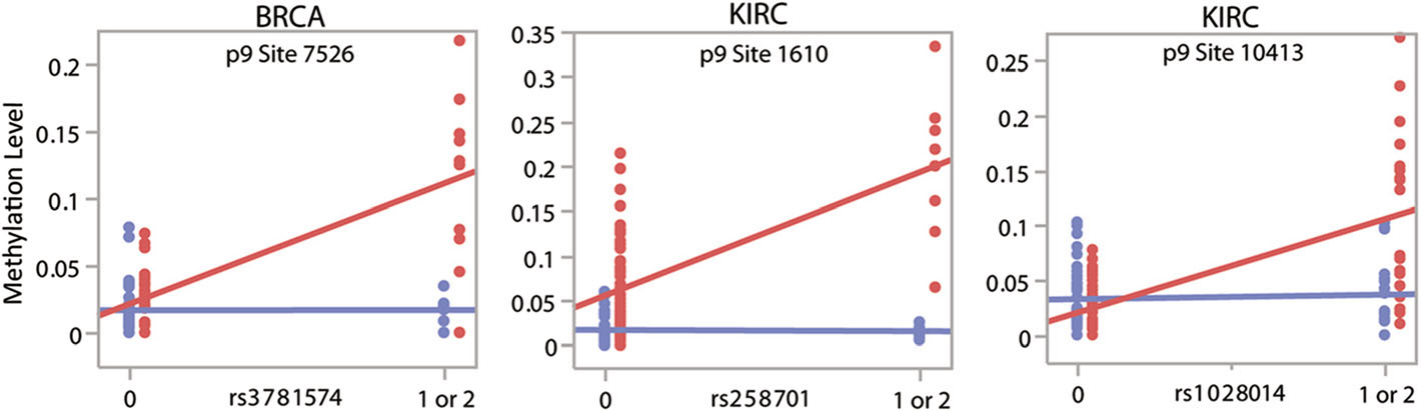
Interaction effects on tRNA p9 site methylation. The three plots show examples of SNPs that have different relationships with the levels of p9 methylation in normal (*blue*) and tumor (*red*) samples

Given the general increase in the levels of p9 methylation in cancers, we assessed whether nuclear genetic variants could modulate the observed changes in mitochondrial RNA processing differentially in tumor relative to normal samples. To do this, we looked for genotype-by-disease-state (tumor or normal) interaction effects on p9 site methylation levels across cancer types. We obtained genotyping data for the same samples for which we measured the levels of p9 site methylation and limited the analysis to SNPs in Hardy–Weinberg equilibrium (*P* > 0.001), those where at least 40 individuals had methylation data available and with at least five individuals carrying a minor allele. Under a dominant model and after filtering SNPs for MAF >5%, this analysis identified 18 peak genotype-by-disease-state interactions at genome-wide significance across cancer types and p9 sites (Table 1; Fig. 3 showing examples). In 15 cases the minor allele is associated with increased levels of p9 site methylation in tumor but not normal samples, suggesting that mitochondrial RNA processing is affected differently in individuals carrying these alleles under cancer status.

**Table 1:** Interaction effects on tRNA p9 site methylation

Cancer	p9 Site	rs number	Chr	Position	Location	Gene	N	MAF	MAF range	*P* value
BRCA	7526	rs3781574	11	33885268	intron	LMO2	46	0.161	0.128–0.231	4.61E-11
BRCA	7526	rs17690328	11	33885390	intron	LMO2	46	0.135	0.016–0.190	1.17E-08
BRCA	7526	rs317391	17	32174605	intron	ASIC2	44	0.085	0.014–0.485	2.46E-09
KIRC	1610	rs258701	7	81761122	intron	CACNA2D1	61	0.058	0.072–0.167	8.27E-10
KIRC	8303	rs10266772	7	14132727	Intergenic	-	61	0.080	0.079–0.254	2.27E-08
KIRC	10413	rs1028014	8	40698232	intron	ZMAT4	64	0.174	0.085–0.236	2.88E-08
KIRC	12146	rs17164416	5	99387447	Intergenic	-	65	0.051	0.000–0.188	9.95E-09
LIHC	12146	rs41465346	1	219059708	Intergenic	-	48	0.052	0.006–0.304	3.73E-08
LIHC	12146	rs16926871	8	62027498	intron	CLVS1	47	0.074	0.038–0.270	1.24E-08
LUAD	585	rs13029285	2	205916078	intron	PARD3B	49	0.057	0.003–0.076	1.81E-10
LUAD	10413	rs7328699	13	28615701	intron	FLT3	46	0.107	0.000–0.124	9.15E-10
LUAD	10413	rs12591927	15	31833911	intron	OTUD7A	44	0.077	0.016–0.289	2.44E-08
LUAD	10413	rs17003208	22	43073031	Intergenic	-	45	0.074	0.096–0.431	1.13E-08
LUAD	10413	rs17003212	22	43079754	Intergenic	-	46	0.063	0.096–0.434	1.18E-11
LUAD	12146	rs4741498	9	15424938	intron	SNAPC3	45	0.063	0.005–0.256	3.68E-08
LUAD	12146	rs7026970	9	15445219	intron	SNAPC3	45	0.063	0.040–0.355	3.68E-08
LUAD	12146	rs7046713	9	15458921	intron	SNAPC3	45	0.063	0.031–0.357	3.68E-08
LUAD	14734	rs341737	8	2782370	Intergenic	-	54	0.107	0.081–0.225	9.86E-09
PRAD	10413	rs7631369	3	1028679	Intergenic	-	48	0.070	0.015–0.418	3.45E-08
PRAD	10413	rs7301597	12	30175147	Intergenic	-	48	0.050	0.000–0.105	3.85E-09
THCA	585	rs17033484	4	156686737	intron	GUCY1B3	54	0.089	0.058–0.187	3.78E-08
THCA	4271	rs2145836	20	47421064	intron	PREX1	44	0.054	0.000–0.058	4.41E-08

*MAF* denotes minor allele frequency of the polymorphism in TCGA data for the specific cancer type, and *MAF range* denotes the range of minor allele frequencies across continents observed in 1000 Genomes phase 3 data [41]

After initial discovery, we attempted to replicate each genome-wide significant interaction effect in other cancer types at any p9 site (*P* < 0.001, under the same criteria outlined above). In doing so, we found an interaction effect for rs317391 in KIRC at p9 site 12146 (*P* = 2.16 × 10^−5^; interaction effect originally observed in BRCA at p9 site 7526, *P* = 2.46 × 10^−9^). This SNP falls within an intron of the gene *ASIC2*, which is part of a sodium channel superfamily. We also found that rs341737 is an interaction SNP for the levels of methylation at p9 site 14734 in PRAD (*P* = 0.000745; interaction effect originally observed in LUAD at p9 site 14734, *P* = 9.86 × 10^−9^). This SNP falls in an intergenic region near to *CMSD1*, a known tumor suppressor gene. The fact that these SNPs are also associated with similar effects in other cancer types suggests that our initial observations are robust.

### Potential clinical implications

**Fig. 4:**
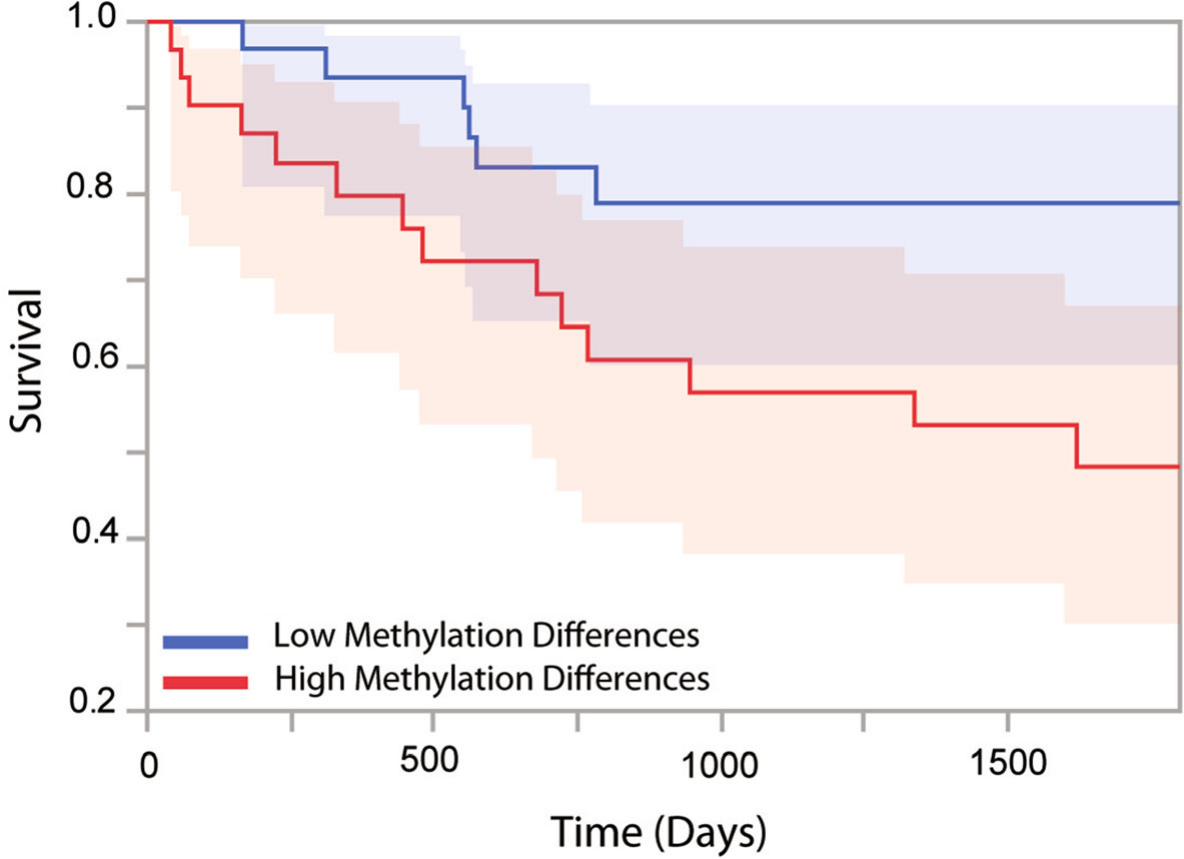
Survival analysis. Data show relationship between the magnitude of change of the levels of methylation between paired tumor and normal samples at p9 site 10413 and survival rates for patients with KIRC

In order to relate changes in mitochondrial RNA processing to potential clinical outcomes we used Cox proportional hazards tests to determine if changes in the levels of p9 site methylation between paired normal and tumor samples are a significant predictor of patient survival outcomes. To ensure power to detect significant associations, we focused on cancers where we had data for at least 50 individuals with >25% death rate within 60 months of diagnosis (only KIRC and LUAD meet this criterion). Treating p9 methylation differences as a quantitative trait we find that methylation differences do not significantly predict patient survival in LUAD. For KIRC, however, seven p9 sites are significant predictors of patient survival at the 5% level, two of which remain significant after Bonferroni correction (Additional file 1: Table S9), with larger increases in the levels of p9 site methylation in tumor compared to paired normal samples being associated with worse survival. The strongest effect occurs for p9 site 10413 (ninth position of *TRNR*; *P* = 0.000476). Repeating the analysis including age, sex, and ethnicity gives similar results: five out of seven remain significant at *P* < 0.05, one out of two remain significant after Bonferroni correction, and the strongest effect for the ninth position of *TRNR* has a *P* value of 0.00151. Treating the change in the levels of methylation at this site as a categorical variable with data binned into two equal sized groups of high and low methylation differences (Fig. 4; *P* = 0.036), the model suggests that those in the larger methylation differences group are 2.784 (95% confidence interval (1.07,7.25)) times more likely to die over a 60-month period after diagnosis.

## Discussion

By analyzing RNA sequencing data across a large number of individuals and sample types, we find significant and consistent changes in the levels of m^1^A and m^1^G post-transcriptional methylation at functionally important positions within mitochondrial tRNAs in tumor samples compared to paired normal samples. These changes appear to be a widespread phenomenon across different types of tumors and suggest that altered mitochondrial RNA processing is a hallmark of cancer. We hypothesize that oncogenic signals trigger these changes, which in turn promote energetic plasticity in tumor cells in a manner that is not dependent on hard-coded mitochondrial genetic changes. The observed positive correlation between p9 site methylation levels and tRNA 5′ cleavage rates highlights the link between the two processes during and/or after transcription of the mitochondrial polycistronic transcript and suggests that methylation is coupled with cleavage. Our analysis also shows that, across different cancers and p9 sites, methylation levels are associated with tRNA/coding gene expression in both positive and negative directions. This suggests that the downstream impacts of changes to p9 site methylation levels on mitochondrial gene expression are complex and may vary both along the polycistronic strand and in different cancer types.

A large number of associations between p9 site methylation levels and the expression of nuclear genes are highly significant in normal tissue, yet these relationships completely break down in tumors. Since associations in normal tissues contain a highly statistically significant enrichment of RNA post-transcriptional modification genes, it is possible that a subset of these genes directly or indirectly affect the levels of p9 site methylation. However, given the large number of observed associations, they also potentially encompass genes whose change in expression is a downstream response to changes in p9 site methylation. Analysis of genes most strongly associated with p9 methylation rates in normal tissues would be a good starting point to tease apart these processes. Mitochondrial tRNAs are key for the translation of genes of the oxidative phosphorylation system [16], and given the connection between cell growth and protein synthesis, it stands to reason that increased rates of tRNA processing would augment the translational and metabolic capacity of mitochondria, modulate the cell cycle, and, ultimately, promote uncontrolled growth. Further studies are warranted to illuminate the molecular mechanisms likely coupling altered levels of mitochondrial RNA processing to cell growth and other tumorigenesis steps such as cell invasion and migration.

The observed differences in the levels of p9 methylation between normal and tumor samples are significant; however, a subset of tumor samples exhibit methylation levels in the range detected in normal samples. The levels of p9 site methylation for these samples are highly predictable from allelic variation, and as such we hypothesize that nuclear genetic variation can modulate an individual’s ability to maintain normal levels of mitochondrial RNA processing during cancer development and subsequently influence patient survival outcomes. Our tests to uncover genotype-by-disease-state interactions revealed 18 peak variants at genome-wide significance and these in vivo interaction effects serve as a promising starting point for a potential method to uncover biomarkers for predictable responses to cancer progression. Although no single interaction should be taken as strong evidence of a role for a given haplotype in tumor development, among the genome-wide significant interaction SNPs that fall within intronic or exonic regions (11 in total) there are several noteworthy examples where these genes may be interesting for further study. Four genes have a link to mitochondrial function, cellular proliferation, or apoptosis (*CACNA2D2*, *CLVS1*, *OTUD7A*, and *FLT3*), three genes have been linked to neurological function (and amyotrophic lateral sclerosis in particular), where dysfunctional mitochondria have a known role [35, 36] (*ASIC2*, *CLVS1*, *PARD3B*) and four genes have been previously linked with cancer (*LMO2*, *CACNA2D2*, *FLT3*, and *PREX1*)*.* These associations were detected through careful statistical analysis and filtering; however, only future functional work would help uncover the mechanisms through which causal variants tagged by these association might modulate p9 site methylation. We note that the MAF range of the 18 SNPs is 0.054–0.174 in TCGA data for the relevant cancer type, but the frequency of these markers vary more wildly across 1000 genome continental groupings (Table 1), suggesting that there may be population differences in the ability to modulate p9 site methylation under cancer status.

Finally, we find that altered mitochondrial tRNA methylation profiles in cancer samples correlate with patient survival outcomes, suggesting that these processes have important downstream consequences and may be clinically relevant. An alternative hypothesis is that other confounding factors strongly linked to survival may influence mitochondrial RNA processing; further analysis with larger sample sizes is required to truly understand these relationships.

## Conclusions

Our study demonstrates that there are major changes to mitochondrial RNA processing in cancers that occur strongly and consistently across different cancer types. We hypothesize that tumor cells use these processes to regulate their mitochondrial output and consequently also the shift between different ways of producing energy and biomaterials in a manner that is not dependent on hard-coded genetic changes. Also, altered mitochondrial RNA processing appears to be due to a breakdown in the pathways involved in RNA modification and processing and may be modulated at least in part by common genetic variation. We also link the extent of changes in mitochondrial processing with patient survival outcomes, thus making a strong case for the prognosis potential of these events in cancer.

Taken as a whole, these results provide strong evidence for altered mitochondrial post-transcriptional modification and processing taking place in cancer and complement an emerging appreciation for the roles that post-transcriptional and post-translational regulation play in the etiology of cancers [37–40]. Although the mechanisms underlying these alterations remain to be resolved, it is tempting to speculate that restoring normal levels of modification and processing of mitochondrial RNA would represent a promising area of investigation for the development of new anticancer therapeutic interventions.

## Additional files

Additional file 1: Figures S1–6 and Tables S1–9. (PDF 1792 kb)

Additional file 2: Table S7. Comparisons between methylation levels at each p9 site with mitochondrial gene expression. Analyses were performed within either normal or tumor samples, and across all cancers. (XLSX 148 kb)

## Abbreviations

BRCA: Breast invasive carcinoma
COAD: Colon adenocarcinoma
HNSC: Head and neck squamous cell carcinoma
KICH: Kidney chromophobe
KIRC: Kidney renal clear cell carcinoma
KIRP: Kidney renal papillary cell carcinoma
LIHC: Liver hepatocellular carcinoma
LUAD: Lung adenocarcinoma
LUSC: Lung squamous cell carcinoma
PRAD: Prostate adenocarcinoma
STAD: Stomach adenocarcinoma
TCGA: The Cancer Genome Atlas
THCA: Thyroid carcinoma
TPM: Transcripts per million
tRNA: Transfer RNA
