# Supplementary material for: Integrated genomic analysis of mitochondrial RNA processing in human cancers
Source: Genome Med. 2017 Apr 18;9:36. doi: 10.1186/s13073-017-0426-0 (PMC5395977; doi:10.1186/s13073-017-0426-0)
Supplement: Supplementary file 1 — Figures S1–6 and Tables S1–9. (PDF 1792 kb) [file 13073_2017_426_MOESM1_ESM.pdf]

Supplementary Materials for:

Title: Integrated Genomic Analysis of Mitochondrial RNA Processing in Human Cancers

Authors: Youssef Idaghdour[1] and Alan Hodgkinson[2]

Affiliations:

1. Biology Program, Division of Science and Mathematics, New York University Abu Dhabi, PO Box 129188, Abu Dhabi, United Arab Emirates.
2. Department of Medical and Molecular Genetics, Guy's Hospital, King's College London, London, SE1 9RT, UK

Content:

Figures S1-6
Supplementary Tables S1-S9

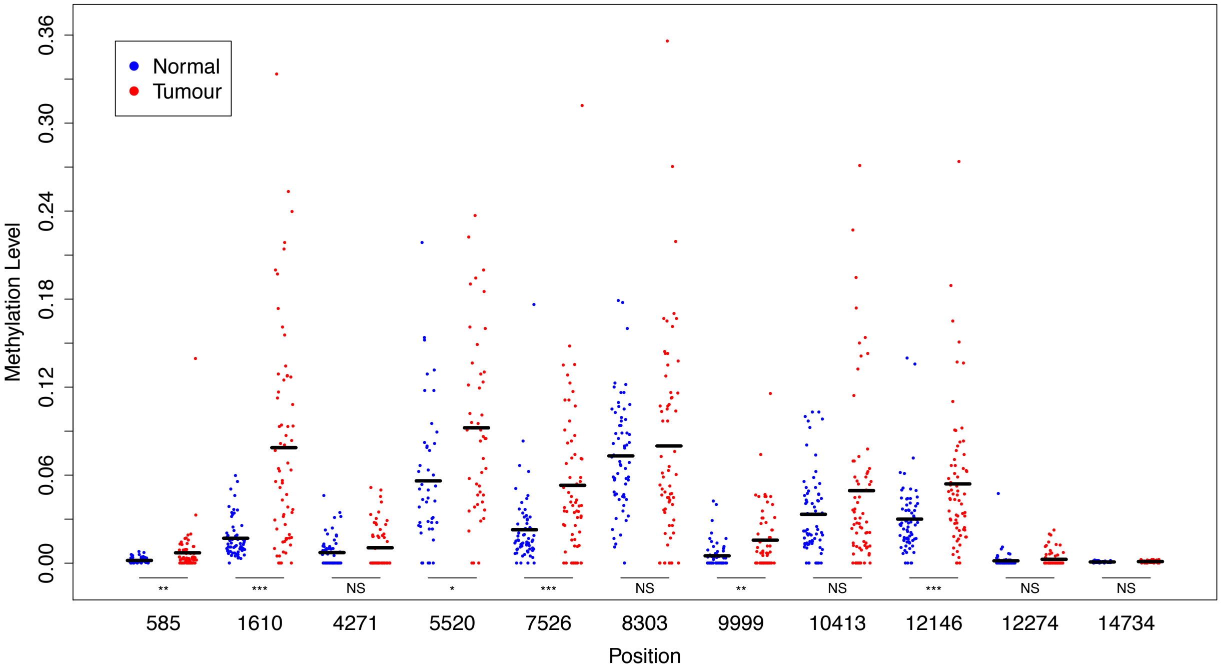

Figure S1: Observed methylation levels for all eleven p9 sites within KIRC, split into normal and tumor. Average p9 methylation levels in each category are shown as horizontal black lines and p-values for differences between paired normal and tumor samples are shown below each p9 site (* for P<0.05, ** for P<0.01, *** for P<0.001 after Bonferroni correction).

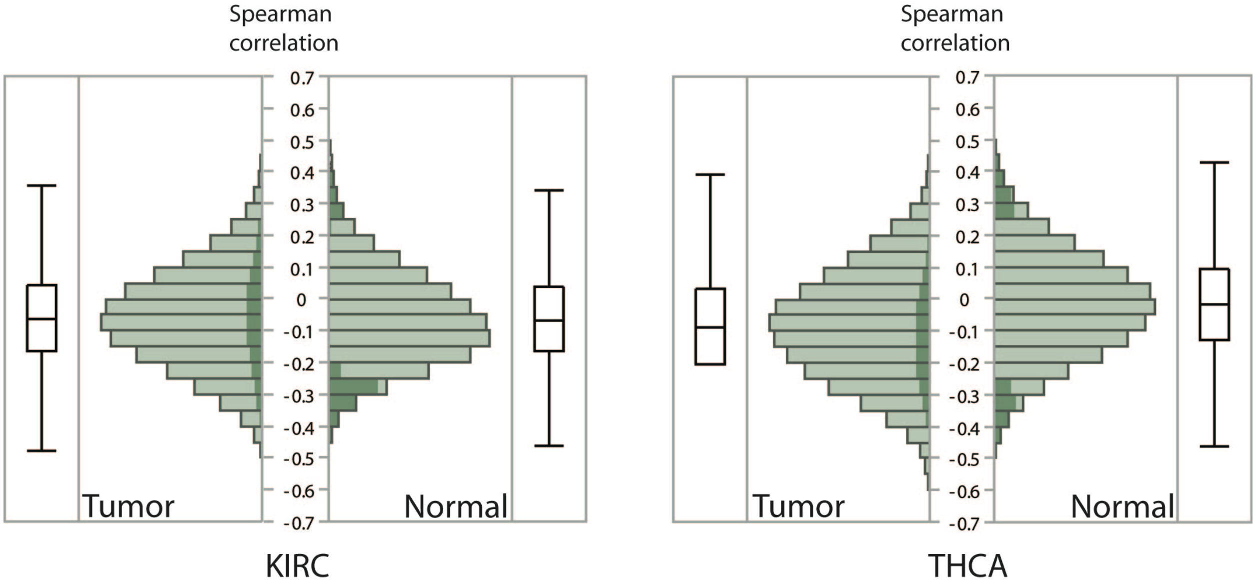

Figure S2: Distribution of Spearman correlations between global gene expression levels and methylation levels at 11 p9 sites in the KIRC (left) and THCA (right) datasets. Associations that are significant at 0.05 p-value threshold in normal samples are highlighted with the dark green color. As in the BRCA dataset (Figure S3), we observe an alteration in the tumor samples of the associations that are significant in the normal samples.

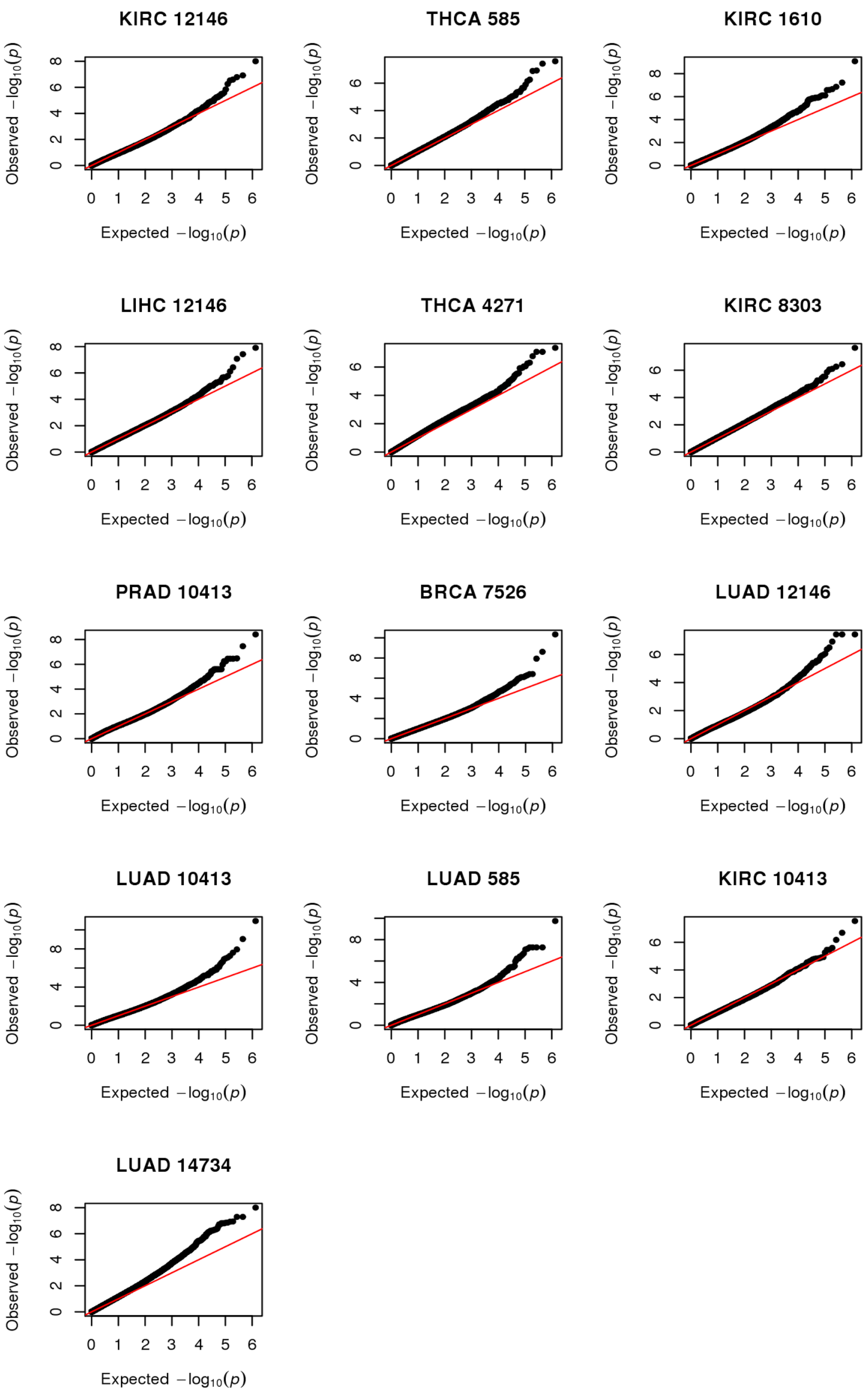

Figure S3: QQ plots for p9 sites and cancers showing significant interaction effects.

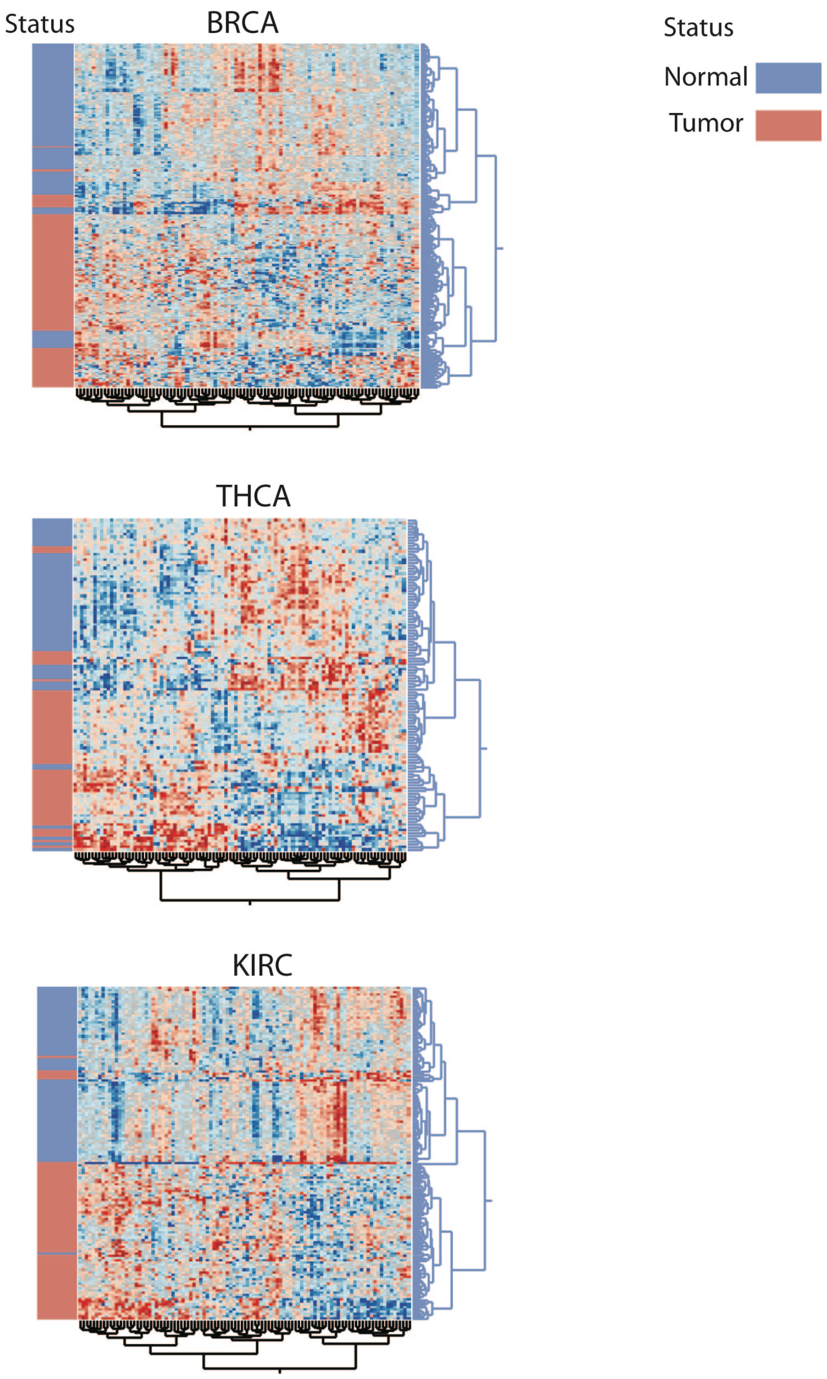

Figure S4: Two-way hierarchical clustering of expression of 99 (100 for THCA) genes encoding mtRNA-binding proteins in BRCA, KIRC and THCA datasets. Each column represents one of the genes and each row represents one individual sample. Sample type is identified by color in the legend to the left of the heat map (normal in blue and tumor in red). Intensity of red indicates high expression relative to the sample mean, and blue relatively low expression. The clustering was generated with the Ward method. The heat map shows the clustering of expression profiles largely by sample type.

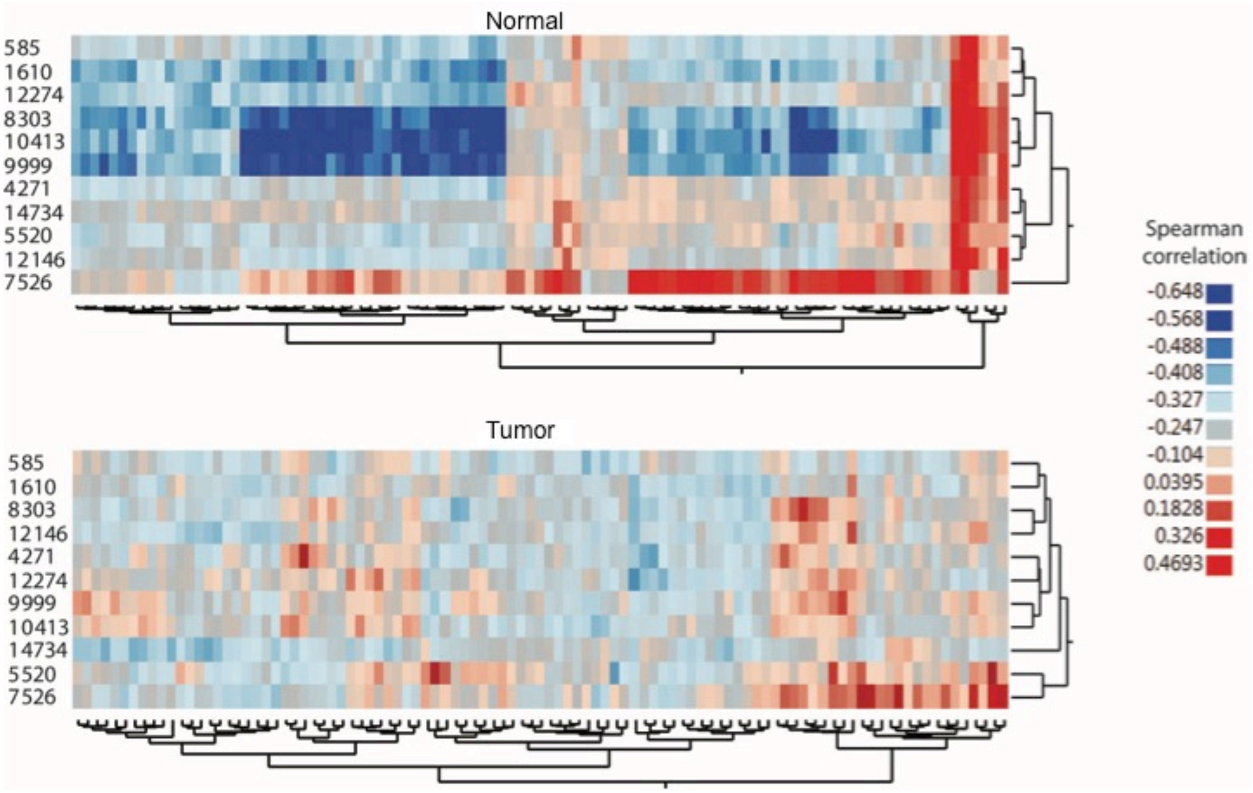

Figure S5: Two-way clustering of Spearman correlations of expression levels of 99 genes encoding mtRNA-binding proteins (columns) and methylation level at 11 p9 sites (rows) in the BRCA dataset. Correlation values are visualized using a red-to-gray-to-blue color theme (values range from 0.62 to -0.66). The clustering was generated with the Ward method.

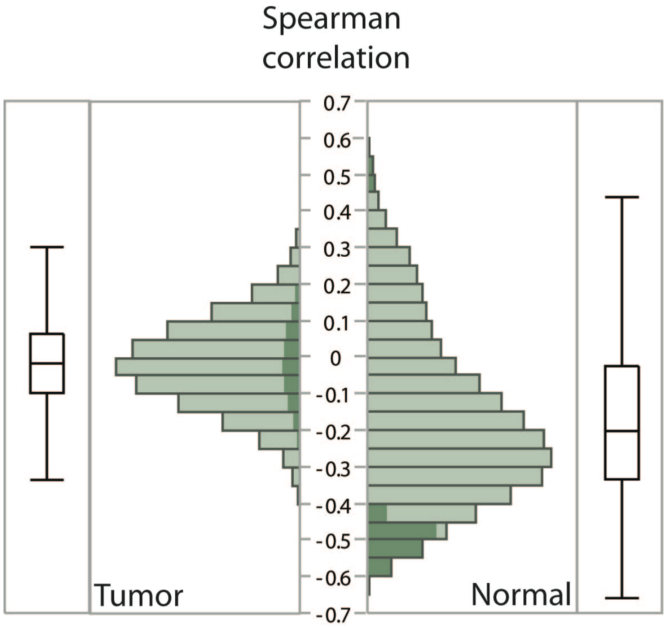

Figure S6: Distribution of Spearman correlations between gene expression levels of 16,736 genes and methylation level at 11 p9 sites in the BRCA dataset. Associations that are significant at Bonferroni threshold in normal samples are highlighted with the dark green color. Note the alteration in the tumor samples of the associations that are significant at Bonferroni threshold in the normal samples.

Table S1: Ingenuity pathway enrichment analysis for BRCA, THCA and KIRC. Nuclear genes whose expression levels are significantly associated with methylation level at p9 sites were subject to the Core Analysis Workflow implemented in the Ingenuity Pathway Analysis package. p9 sites were included for BRCA if >50 genes showed significant correlations, and for THCA and KIRC, site 10413 was used for replication of BRCA results.

Cancer	Site	Functional Category	P-value Range	Molecules
BRCA	585	Cell-to-Cell Signalling and Interaction	$3.61e^{-2} – 6.00e^{-6}$	18
		Cellular Movement	$3.61e^{-2} – 3.31e^{-4}$	38
		Gene Expression	$3.61e^{-2} – 5.06e^{-4}$	62
		Cell Death and Survival	$3.61e^{-2} – 6.11e^{-4}$	44
		Cellular Assembly and Organisation	$3.61e^{-2} – 8.54e^{-4}$	42
BRCA	1610	Cellular Assembly and Organisation	$4.22e^{-2} – 1.88e^{-4}$	87
		Cell-to-Cell Signalling and Interaction	$4.22e^{-2} – 7.49e^{-4}$	31
		Cellular Development	$4.30e^{-2} – 1.07e^{-3}$	50
		Cell Signalling	$4.28e^{-2} – 1.13e^{-3}$	32
		Post-translational Modification	$4.71e^{-2} – 1.13e^{-3}$	57
BRCA	8303	RNA Post-Transcriptional Modification	$3.04e^{-2} – 1.17e^{-19}$	118
		DNA Replication, Recombination and Repair	$4.58e^{-2} – 2.00e^{-10}$	231
		Cell Cycle	$4.58e^{-2} – 4.58e^{-7}$	162
		Cellular Growth and Proliferation	$4.58e^{-2} – 4.97e^{-5}$	719
		Protein Synthesis	$1.04e^{-2} – 5.48e^{-5}$	168
BRCA	9999	RNA Post-Transcriptional Modification	$4.50e^{-3} – 3.54e^{-30}$	166
		Gene Expression	$3.91e^{-2} – 9.27e^{-13}$	718
		DNA Replication, Recombination and Repair	$4.99e^{-2} – 3.24e^{-9}$	363
		Cell Cycle	$4.99e^{-2} – 5.64e^{-8}$	478
		Post-translational Modification	$3.33e^{-2} – 3.53e^{-5}$	35
BRCA	12274	RNA Post-Transcriptional Modification	$3.33e^{-2} – 4.39e^{-13}$	33
		Protein Synthesis	$3.54e^{-2} – 1.52e^{-12}$	55
		Gene Expression	$3.33e^{-2} – 2.18e^{-11}$	30
		Cell Cycle	$3.60e^{-2} – 3.61e^{-4}$	29
		DNA Replication, Recombination and Repair	$4.17e^{-2} – 3.61e^{-4}$	20
BRCA	10413	RNA Post-Transcriptional Modification	$2.77e^{-2} – 3.27e^{-31}$	139
		DNA Replication, Recombination and Repair	$4.79e^{-2} – 2.45e^{-13}$	241
		Cell Cycle	$4.79e^{-2} – 2.83e^{-7}$	268
		Gene Expression	$4.09e^{-2} – 1.53e^{-4}$	423
		Cellular Compromise	$3.54e^{-2} – 3.62e^{-4}$	19
KIRC	10413	RNA Post-Transcriptional Modification	$3.60e^{-2} – 9.50e^{-4}$	23
		Cell Death and Survival	$3.98e^{-2} – 1.33e^{-3}$	153
		Cell Cycle	$3.98e^{-2} – 1.58e^{-3}$	20
		Gene Morphology	$3.98e^{-2} – 1.58e^{-3}$	40
		Cell-to-Cell Signalling and Interaction	$3.98e^{-2} – 1.58e^{-3}$	17
THCA	10413	Cellular Growth and Proliferation	$3.33e^{-2} – 3.42e^{-6}$	194
		DNA Replication, Recombination and Repair	$4.10e^{-2} – 1.36e^{-5}$	63
		Gene Expression	$3.17e^{-2} – 3.37e^{-5}$	110
		Protein Synthesis	$3.21e^{-2} – 3.37e^{-5}$	48
		RNA Post-Transcriptional Modification	$3.05e^{-2} – 1.05e^{-4}$	28

Table S2: Differences in methylation level in normal and tumor samples at each p9 site in each cancer type. The table shows the number of paired samples used in each test (N), the fold change in tumor samples compared to normal samples (F) and the P value for a paired Wilcoxon signed-rank test (P).

	**P9 Sites**										
	**585**	**1610**	**4271**	**5520**	**7526**	**8303**	**9999**	**10413**	**12146**	**12274**	**14734**
**BRCA N**	108	91	90	58	56	95	107	108	108	109	109
**BRCA F**	1.19	2.17	1.68	2.11	3.06	1.16	1.02	0.93	1.60	0.65	1.04
**BRCA P**	9.9E-01	3.0E-03	9.2E-02	3.9E-05	2.1E-05	2.4E-01	7.8E-01	9.2E-01	1.9E-01	1.6E-01	6.5E-01
**COAD N**	23	18	15	4	13	21	21	20	23	23	25
**COAD F**	2.07	1.85	1.94	6.81	2.28	0.69	0.36	0.27	0.40	0.38	1.29
**COAD P**	2.5E-01	4.2E-02	3.3E-01	1.3E-01	9.2E-02	6.0E-02	9.3E-02	4.6E-03	1.1E-03	3.1E-01	2.3E-01
**HNSC N**	38	27	24	12	16	33	39	38	40	41	42
**HNSC F**	1.62	2.02	2.05	1.45	2.86	0.75	1.97	0.73	0.81	5.24	1.43
**HNSC P**	6.6E-01	1.5E-01	2.9E-01	3.8E-01	2.1E-02	1.5E-01	6.4E-02	3.5E-01	1.8E-01	2.9E-01	8.3E-01
**KICH N**	24	24	20	16	17	24	24	24	24	24	24
**KICH F**	17.78	6.50	1.81	2.70	4.23	4.27	6.84	3.95	3.84	2.31	1.76
**KICH P**	1.3E-05	8.3E-06	2.2E-01	1.7E-03	3.8E-02	8.3E-06	2.3E-03	3.5E-04	1.2E-03	2.0E-02	5.3E-03
**KIRC N**	70	64	59	46	63	64	61	67	68	69	70
**KIRC F**	3.83	4.64	1.46	1.64	2.33	1.09	3.12	1.48	1.80	1.63	1.31
**KIRC P**	1.3E-04	4.4E-09	1.9E-01	1.6E-03	7.5E-06	9.7E-01	6.0E-04	1.2E-01	5.6E-05	8.8E-02	2.0E-02
**KIRP N**	32	25	28	26	26	32	32	32	32	32	32
**KIRP F**	5.14	6.16	0.97	1.57	4.51	1.16	1.71	1.31	1.51	1.44	1.71
**KIRP P**	5.6E-03	4.5E-05	5.4E-01	4.3E-02	1.1E-05	4.5E-01	6.5E-01	3.0E-01	3.2E-01	5.4E-01	3.2E-01
**LIHC N**	41	28	43	36	34	50	50	50	50	50	50
**LIHC F**	2.11	2.92	2.21	1.41	2.17	1.61	1.47	1.44	1.49	2.12	1.19
**LIHC P**	3.2E-01	2.2E-03	4.8E-03	2.3E-02	5.4E-02	4.6E-03	2.0E-01	2.1E-01	7.7E-02	1.3E-01	5.4E-01
**LUAD N**	54	36	25	12	29	36	41	47	46	50	56
**LUAD F**	1.94	2.06	2.18	2.31	2.81	1.47	1.23	1.35	1.37	1.04	1.01
**LUAD P**	3.0E-01	2.5E-02	4.7E-02	2.3E-01	1.0E-02	4.1E-01	3.0E-01	7.8E-01	6.4E-01	9.1E-01	3.0E-01
**LUSC N**	47	36	35	9	25	46	49	49	49	50	51
**LUSC F**	1.39	1.35	1.15	0.84	3.75	0.77	0.89	0.82	0.92	0.97	1.42
**LUSC P**	7.7E-01	5.4E-02	5.6E-01	2.9E-01	5.9E-03	5.6E-02	2.2E-01	3.2E-02	5.8E-01	5.2E-02	9.4E-01
**PRAD N**	49	43	33	17	38	45	50	50	48	50	50
**PRAD F**	0.94	0.71	1.39	1.34	0.73	1.14	0.49	0.47	0.78	0.43	0.84
**PRAD P**	2.2E-01	9.0E-03	7.6E-01	1.4E-01	4.7E-02	3.1E-01	1.6E-01	1.3E-02	5.9E-01	4.1E-02	3.0E-01
**STAD N**	30	31	31	25	23	20	30	29	31	29	31
**STAD F**	2.89	2.00	1.52	1.97	2.85	1.90	2.80	1.38	0.95	0.87	1.11
**STAD P**	9.1E-03	1.4E-02	9.0E-02	2.5E-02	6.4E-03	1.1E-01	3.3E-02	2.7E-01	4.8E-01	3.2E-01	3.0E-01
**THCA N**	57	36	46	39	47	57	57	57	57	57	57
**THCA F**	0.47	1.56	1.65	2.72	2.88	1.21	1.99	1.57	2.75	1.23	1.05
**THCA P**	1.2E-02	1.5E-01	2.9E-01	1.3E-04	2.2E-05	2.2E-01	9.1E-01	5.6E-02	2.1E-05	6.1E-01	5.8E-01

Table S3: Differences in methylation level in normal and tumor samples at each p9 site in each cancer type after resampling. Data was resampled within each individual to the lowest coverage found in either the normal or tumor sample for each given position. The table shows the number of paired samples used in each test (N), the fold change in tumor samples compared to normal samples (F) and the P value for a paired Wilcoxon signed-rank test (P).

	**P9 Sites**										
	**585**	**1610**	**4271**	**5520**	**7526**	**8303**	**9999**	**10413**	**12146**	**12274**	**14734**
**BRCA N**	108	91	90	58	56	95	107	108	108	109	109
**BRCA F**	0.94	2.10	1.95	2.04	2.32	1.11	1.04	0.91	1.59	0.54	1.01
**BRCA P**	8.1E-01	4.7E-03	3.3E-02	2.3E-04	4.1E-04	4.0E-01	6.1E-01	8.7E-01	7.7E-02	6.7E-02	6.7E-01
**COAD N**	23	18	15	4	13	21	21	20	23	23	25
**COAD F**	1.74	2.03	1.77	7.32	2.15	0.69	0.23	0.33	0.40	0.34	0.90
**COAD P**	3.7E-01	5.2E-02	2.6E-01	1.3E-01	2.0E-01	5.1E-02	2.5E-02	1.0E-02	3.5E-03	1.4E-01	1.0E+00
**HNSC N**	38	27	24	12	16	33	39	38	40	41	42
**HNSC F**	1.89	2.25	1.53	2.36	2.99	0.71	1.86	0.60	0.89	6.18	1.65
**HNSC P**	4.7E-01	2.2E-02	5.6E-01	1.3E-01	6.0E-02	1.7E-01	8.7E-02	1.2E-01	3.3E-01	2.2E-01	2.9E-01
**KICH N**	24	24	20	16	17	24	24	24	24	24	24
**KICH F**	9.77	6.32	1.10	2.77	4.99	4.36	7.44	4.50	3.83	2.07	1.59
**KICH P**	1.1E-04	1.6E-05	8.6E-01	6.9E-03	1.4E-02	3.0E-06	2.1E-03	4.0E-04	9.6E-04	1.3E-01	7.2E-02
**KIRC N**	70	64	59	46	63	64	61	67	68	69	70
**KIRC F**	4.88	4.47	1.67	1.67	2.30	1.12	3.64	1.41	1.76	2.01	1.53
**KIRC P**	1.2E-05	6.2E-09	1.7E-01	1.2E-03	2.5E-05	6.2E-01	6.9E-04	1.7E-01	6.2E-04	5.5E-02	1.6E-03
**KIRP N**	32	25	28	26	26	32	32	32	32	32	32
**KIRP F**	4.98	5.73	1.56	1.60	4.78	1.09	1.28	1.12	1.26	0.92	1.38
**KIRP P**	2.0E-03	1.8E-04	8.1E-01	3.1E-02	3.7E-04	7.6E-01	7.3E-01	8.3E-01	5.2E-01	5.1E-01	5.2E-01
**LIHC N**	41	28	43	36	34	50	50	50	50	50	50
**LIHC F**	2.39	3.32	2.40	1.42	2.55	1.80	1.57	1.40	1.45	1.57	1.24
**LIHC P**	4.2E-01	8.8E-04	1.3E-02	1.8E-02	2.2E-02	2.0E-03	1.7E-01	2.9E-01	8.1E-02	2.5E-01	3.3E-01
**LUAD N**	54	36	25	12	29	36	41	47	46	50	56
**LUAD F**	2.65	2.31	2.17	1.91	2.50	1.32	1.27	1.34	1.25	0.86	0.99
**LUAD P**	6.0E-02	1.1E-02	6.9E-02	3.1E-01	3.0E-02	7.3E-01	4.9E-01	9.2E-01	9.8E-01	8.1E-01	6.5E-01
**LUSC N**	47	36	35	9	25	46	49	49	49	50	51
**LUSC F**	1.88	1.40	1.11	0.91	3.83	0.75	0.75	0.85	0.89	1.25	1.31
**LUSC P**	2.7E-01	4.3E-02	5.1E-01	6.7E-01	4.3E-03	7.8E-02	1.0E-01	1.0E-01	9.4E-01	5.6E-01	4.3E-01
**PRAD N**	49	43	33	17	38	45	50	50	48	50	50
**PRAD F**	1.07	0.72	1.43	1.64	0.84	1.16	0.51	0.49	0.74	0.33	0.79
**PRAD P**	9.5E-01	5.2E-02	8.0E-01	7.9E-02	3.8E-01	3.0E-01	2.5E-01	1.5E-02	6.2E-01	7.9E-03	1.7E-01
**STAD N**	30	31	31	25	23	20	30	29	31	29	31
**STAD F**	3.26	1.93	1.45	2.30	3.32	1.93	3.23	1.33	0.97	0.76	1.38
**STAD P**	3.9E-03	2.2E-02	1.5E-01	1.3E-02	4.0E-03	1.1E-01	5.4E-02	3.6E-01	5.6E-01	4.3E-01	3.4E-02
**THCA N**	57	36	46	39	47	57	57	57	57	57	57
**THCA F**	0.47	1.68	2.17	2.52	3.27	1.21	2.38	1.57	2.73	1.07	0.97
**THCA P**	2.9E-02	6.7E-02	2.7E-01	5.0E-04	2.1E-05	2.8E-01	9.4E-01	7.0E-02	1.1E-04	4.7E-01	9.3E-01

Table S4: Differences in methylation levels in normal and tumor samples at each p9 site across all cancer types combined. For each p9 site methylation level data was standardized within each cancer (thus maintaining cancer associated patterns in methylation rates). In total, 5 out of the 11 sites show Bonferroni significant differences using two-sided paired Wilcoxon signed-rank tests.

p9 Site	Prob>|S|	Bonferroni Significant
**585**	0.0416	No
**1610**	1.6017E-18	Yes
**4271**	0.0014	Yes
**5520**	5.0351E-17	Yes
**7526**	1.0992E-20	Yes
**8303**	0.0268	No
**9999**	0.0482	No
**10413**	0.7445	No
**12146**	0.0014	Yes
**12274**	0.3202	No
**14734**	0.0489	No

Table S5: **Comparisons between p9 methylation levels and the rate of cleavage occurring at the 5' end of each respective tRNA, which we measured as the proportion of sequencing reads starting or ending either side of this position (observed).** As a control, we also considered cleavage rates a further 9bp upstream from each p9 site (control). Sites were considered if they show significant differences in methylation between normal and tumor samples at the 5% significance level.

Cancer	P9 Site	Sample Pairs	P-value (observed)	R (observed)	P-value (control)	R (control)
BRCA	1610	91	1.8E-01	0.10	91	8.9E-01
BRCA	5520	42	3.3E-04	0.38	42	2.1E-01
BRCA	7526	56	8.5E-01	0.02	56	3.9E-01
COAD	10413	20	2.1E-03	0.47	20	1.0E+00
COAD	12146	23	3.4E-01	0.14	23	9.5E-01
KICH	585	23	5.7E-01	0.09	23	1.1E-01
KICH	1610	24	2.0E-04	0.51	24	9.9E-01
KICH	8303	24	4.4E-03	0.40	24	3.8E-01
KICH	9999	24	2.1E-02	0.33	24	2.3E-01
KICH	10413	24	4.7E-04	0.49	24	1.8E-01
KICH	12146	24	1.3E-06	0.63	24	2.9E-01
KICH	12274	24	5.3E-01	0.09	24	6.7E-01
KICH	14734	24	5.6E-01	-0.09	24	8.8E-04
KIRC	585	70	3.1E-01	-0.09	70	5.1E-01
KIRC	1610	64	4.5E-06	0.39	64	7.1E-03
KIRC	5520	29	8.0E-01	0.03	29	5.0E-01
KIRC	7526	62	3.5E-01	0.08	62	8.6E-01
KIRC	9999	61	8.6E-01	0.02	61	2.2E-01
KIRC	12146	67	5.6E-06	0.38	67	2.9E-01
KIRC	14734	70	7.0E-01	0.03	70	6.6E-02
KIRP	585	30	9.6E-01	0.01	30	8.4E-01
KIRP	1610	25	6.7E-03	0.38	25	3.7E-02
KIRP	7526	26	8.1E-05	0.52	26	2.9E-01
LIHC	1610	28	4.0E-01	-0.11	28	7.6E-02
LIHC	4271	43	5.7E-02	0.21	43	8.1E-01
LIHC	8303	50	6.3E-02	-0.19	50	3.7E-01
LUAD	1610	36	6.9E-02	0.22	36	9.8E-02
LUAD	4271	24	9.5E-01	0.01	24	5.1E-02
LUAD	7526	29	8.5E-02	0.23	29	3.3E-01
LUSC	7526	25	8.3E-01	0.03	25	7.0E-01
LUSC	10413	49	1.0E-04	0.38	49	5.5E-02
PRAD	1610	43	8.0E-01	-0.03	43	7.3E-01
PRAD	7526	38	6.0E-01	0.06	38	3.1E-02
PRAD	10413	50	2.7E-05	0.41	50	1.4E-01
PRAD	12274	50	9.6E-01	-0.01	50	4.6E-01
STAD	585	30	4.1E-02	0.27	30	9.1E-01
STAD	1610	31	2.4E-01	0.15	31	3.7E-02
STAD	5520	23	2.5E-01	0.17	23	3.6E-01
STAD	7526	22	6.0E-01	0.08	22	2.5E-01
STAD	9999	30	4.1E-01	0.11	30	2.4E-01
THCA	585	56	3.2E-01	-0.09	56	5.7E-01
THCA	7526	46	5.9E-01	0.06	46	6.2E-01
THCA	12146	57	6.3E-03	0.25	57	5.9E-01

Table S6: **Comparisons between p9 methylation levels and tRNA expression levels.**

Cancer	P9 site	Samples	P value	R
BRCA	1610	188	2.25E-02	0.166
BRCA	5520	122	6.12E-02	-0.170
BRCA	7526	118	5.72E-03	-0.253
COAD	1610	36	5.77E-01	0.096
COAD	10413	40	2.31E-02	-0.359
COAD	12146	46	9.02E-03	-0.381
HNSC	7526	32	1.98E-02	-0.410
KICH	585	48	1.21E-05	-0.586
KICH	1610	48	4.38E-01	0.114
KICH	5520	32	2.09E-02	0.407
KICH	7526	34	2.82E-03	0.496
KICH	8303	48	4.92E-03	0.399
KICH	9999	48	7.06E-01	0.056
KICH	10413	48	7.18E-01	-0.053
KICH	12146	48	1.07E-05	-0.589
KICH	12274	48	1.13E-01	-0.232
KICH	14734	50	9.22E-01	0.014
KIRC	585	144	4.83E-02	-0.165
KIRC	1610	132	3.07E-07	-0.428
KIRC	5520	96	7.83E-01	0.028
KIRC	7526	130	3.39E-02	-0.186
KIRC	9999	126	7.62E-01	-0.027
KIRC	12146	140	3.17E-06	-0.382
KIRC	14734	144	2.38E-01	0.099
KIRP	585	64	2.56E-01	-0.144
KIRP	1610	50	4.21E-01	-0.116
KIRP	5520	52	1.81E-01	-0.188
KIRP	7526	52	4.91E-03	-0.384
LIHC	1610	56	3.54E-02	0.282
LIHC	4271	86	1.09E-01	0.174
LIHC	5520	72	2.52E-01	0.137
LIHC	8303	100	3.01E-02	0.217
LUAD	1610	74	5.99E-01	0.062
LUAD	4271	52	5.23E-03	0.382
LUAD	7526	58	6.29E-01	-0.065
LUSC	7526	50	9.53E-01	-0.009
LUSC	10413	98	2.32E-01	-0.122
PRAD	1610	88	4.32E-01	0.085
PRAD	7526	78	1.13E-02	-0.285
PRAD	10413	102	2.77E-03	-0.293
PRAD	12274	102	9.98E-01	0.000
STAD	585	66	8.50E-03	-0.321
STAD	1610	68	2.04E-01	-0.156
STAD	5520	54	9.30E-01	0.012
STAD	7526	52	4.38E-01	0.110
STAD	9999	66	6.59E-01	0.055
THCA	585	118	8.69E-01	-0.015
THCA	5520	82	1.06E-02	-0.281
THCA	7526	98	2.77E-03	-0.299
THCA	12146	118	2.42E-08	-0.486

Table S7: Comparisons between methylation levels at each p9 site with mitochondrial gene expression. Analyses are performed within either normal or tumor samples, and across all cancers.

Table available as additional file 2

Table S8: Number of significant cross-correlation associations at Bonferroni threshold (p-value < 0.000003). Association tests were performed between nuclear gene expression (16,736 genes) and methylation levels at 11 p9 sites in the BRCA dataset.

P9 Site	BRCA Normal	BRCA Tumor
**585**	397	0
**1610**	1318	0
**4271**	0	0
**5520**	0	0
**7526**	0	0
**8303**	4109	0
**9999**	5829	0
**10413**	6061	0
**12146**	26	0
**12274**	752	1
**14734**	1	1

Table S9: Results from survival analysis for kidney renal clear cell carcinoma. One model was ran where the difference in methylation levels at p9 sites between normal and tumor were used as a quantitative trait, and a second model was ran where methylation differences were binned into two equal sized groups. Within each case, tests were ran with and without covariates (age, gender and ethnicity).

**P9 Sites**	**Quantitative Model**		**Categorical Model**			**Sample Size**	**Deaths**
	P-value covariates	P-value	P-value covariates	P-value	Hazard Ratio		
585	0.00535	0.00107	0.0691	0.133	2.0289	67	20
1610	0.0568	0.0257	0.137	0.127	2.2527	61	17
4271	0.138	0.102	0.138	0.116	2.1123	56	19
5520	0.05	0.311	0.0476	0.0846	3.1594	43	12
7526	0.0186	0.00883	0.067	0.0592	2.734	60	17
8303	0.0575	0.0307	0.829	0.967	1.01924	61	19
9999	0.0286	0.0266	0.0754	0.116	2.1745	58	19
10413	0.00151	0.000476	0.054	0.036	2.7844	64	20
12146	0.116	0.0243	0.0394	0.0252	3.1842	65	20
12274	0.2664	0.24	0.9896	0.753	0.8684	66	20
14734	0.8613	0.787	0.9711	0.922	1.04512	67	20
